# Level of PD-1 expression on CD8+ T cells influence prognosis and respond to PD-1 therapy in a murine model

**DOI:** 10.1186/2051-1426-3-S2-P228

**Published:** 2015-11-04

**Authors:** Benjamin A Kansy, Raghvendra M Srivastava, Hyun-Bae Jie, Gulidanna Shayan, Yu Lei, Jing Li, William Gooding, Sven Brandau, Stephan Lang, Nicole Schmitt, Gordon J Freeman, David A Clump, Robert L Ferris

**Affiliations:** 1 Department of Otorhinolaryngology, University Hospital Essen, Germany; University of Pittsburgh Cancer Institute, Pittsburgh, PA, USA; 2 University of Pittsburgh Cancer Institute, Pittsburgh, PA, USA; 3 Biostatistics Facility, University of Pittsburgh Cancer Institute, Pittsburgh, PA, Pittsburgh, PA, USA; 4 Department of Otorhinolaryngology, University Hospital Essen, Germany, Essen, Germany; 5 Department of Otolaryngology, University of Pittsburgh, Pittsburgh, PA, Pittsburgh, PA, USA; 6 Dana-Farber/Harvard Cancer Center, Boston, MA, USA; 7 Department of Radiation Oncology, University of Pittsburgh Cancer Institute, Pittsburgh, PA, USA

## Introduction

Prognosis varies dramatically in head and neck squamous cell carcinoma (HNSCC) based on HPV status. In HPV^+^ patients, programmed death (PD)-1 expression has been linked to a better clinical outcome. We hypothesized that extent of PD-1 expression may differentially impact T cell phenotype, patient prognosis and response to anti-PD-1 immunotherapy in a murine HPV^+^ cancer model.

## Material and methods

Freshly isolated tumor infiltrating lymphocytes (TIL) from HNSCC patients were stained by flow cytometry for expression level of PD-1 expression (PD-1^high^ vs. PD-1^low^), granzyme B, or IFN-y secretion by ELISPOT. The prognostic impact of PD-1^high^ vs. PD-1^low^ T cells was determined in a cohort of HNSCC patients (n=56, median follow up=19 mo). In a murine HNSCC model, PD-1^high^ and PD-1^low^ fractions were compared from CD3^+^ CD8^+^ PD-1^+^ cells and analyzed according to different treatment groups (untreated, anti-PD-1 mAb, radiotherapy and 3 different anti-PD-1/radiotherapy combinations).

## Results

CTLA-4 and PD-1 were significantly upregulated on both HPV^+^ and HPV^-^ HNSCC patients' TIL, whereas PD-1^+^ CD8^+^ cells were significantly enriched in TIL from HPV^+^ patients (p=0.006). Interestingly, PD-1^high^ cells represented a more dysfunctional phenotype, with severely compromised IFN-γ secretion (phigh CD8^+^ TIL were more likely to be HPV^-^ and had a worse disease free survival (HR = 2.25; 95% CI = 1.46 – 3.15; p < .0001), while high fractions of PD-1^low^ T cells were associated with better clinical outcome (HR= 0.19, 95% CI = .07 - .49, p = .0006), and were seen preferentially in HPV^+^ HNSCC patients. In the murine HNSCC model, anti-PD-1 mAb plus radiotherapy resulted in optimal tumor elimination, which was associated with an elimination of PD-1^high^ CD8^+^ T cells and – most importantly – increase in PD-1^low/intermed^ T cell frequencies (p < 0.5).

## Discussion

Consideration of different PD-1 expression levels on TIL segregates PD-1 expression as a marker of activated, competent tumor reactive T cells on the one hand (low/intermed expression), and as a marker of exhausted, dysfunctional cells in the tumor microenvironment on the other hand (high expression). These results emphasize the crucial role of differential PD-1 expression levels on HNSCC patients' effector T cells for prognosis and as a potential novel biomarker for anti-PD-1/PD-L1 based immunotherapy.

